# Novel variant in *PNPLA6* gene causes Oliver-McFarlane syndrome in a Chinese family: 13 years follow-up 

**DOI:** 10.3389/fgene.2025.1693876

**Published:** 2025-11-14

**Authors:** Panpan Xiao, Yonghua Gu, Xiaolong Qi, Ting Li, Tingting Zuo, Yule Xie, Shuang Zhang, Xunlun Sheng

**Affiliations:** 1 People’s Hospital of Ningxia Hui Autonomous Region, Ningxia Medical University, Yinchuan, China; 2 Ningxia Eye Hospital, People’s Hospital of Ningxia Hui Autonomous Region, Yinchuan, China; 3 Refractive Surgery Department, Gansu Aier Ophthalmology and Optometry Hospital, Lanzhou, China

**Keywords:** PNPLA6, Oliver-McFarlane syndrome, trichomegaly, multiple pituitary hormone deficiencies, chorioretinal dystrophy, night blindness, retinitis pigmentosa

## Abstract

**Introduction:**

Oliver-McFarlane syndrome (OMCS) is a rare autosomal recessive disorder characterized by trichomegaly, severe chorioretinal dystrophy, and multiple pituitary hormone deficiencies. Its marked genetic and clinical heterogeneity presents significant challenges for definitive diagnosis.

**Methods:**

In this study, we initially evaluated a proband clinically diagnosed with OMCS, followed by genetic analysis using whole-exome sequencing (WES). Candidate pathogenic variants were validated via Sanger sequencing and familial co-segregation analysis.

**Results:**

WES identified compound heterozygous variants in the *PNPLA6* gene: a known missense variant (c.3241G>A, p.Gly1081Arg) and a novel missense variant (c.3461G>A, p.Arg1154His). Over a 13-year follow-up, multisystem involvement was observed, including progressive retinochoroidopathy, trichomegaly, growth retardation, and intellectual disability. Disease progression was evident, with severe exacerbation of retinochoroidopathy accompanied by newly developed pituitary hormone deficiencies and absent secondary sexual characteristics.

**Discussion:**

Our findings expand the pathogenic variant spectrum and clinical phenotypic landscape of OMCS. Given the early onset and progressive nature of retinal involvement, we propose that early intervention targeting the preservation of retinal pigment epithelium (RPE) and photoreceptor function may be clinically beneficial.

## 1 Introduction

Oliver-McFarlane syndrome (OMCS; MIM #275400) is a rare autosomal recessive disorder characterized by congenital trichomegaly, severe chorioretinal dystrophy, and multiple pituitary hormone deficiencies-including growth hormone (GH), gonadotropins (luteinising hormone, [LH] and follicle-stimulating hormone [FSH]), and thyroid-stimulating hormone (TSH) (Lisbje et al., 2021). With an estimated prevalence of <1/1,000,000, affected individuals typically present with congenital trichomegaly and elongated eyebrows, develop night blindness and visual impairment before age 5, and exhibit progressive endocrine deficits during adolescence (Lisbje et al., 2021; Hufnagel et al., 2015). These endocrine abnormalities lead to intellectual disability, visual impairment, delayed bone age, and growth retardation, underscoring the need for specific diagnosis in patients with multisystem involvement.

Historically, OMCS diagnosis relied on clinical presentation. Advances in genetic sequencing have revealed that all reported OMCS cases arise from pathogenic variants in *PNPLA6 gene* (Patatin-like phospholipase domain-containing 6; MIM *603197). This gene encodes neuropathy target esterase (NTE) and is associated with a phenotypic continuum encompassing several syndromes, including: Boucher-Neuhauser syndrome (BNS; MIM #215470), Gordon-Holmes syndrome (GHS; MIM #212840), Oliver-McFarlane syndrome (OMCS; MIM,#275400), Lawrence-moon syndrome (LMS; MIM #245800) and spastic paraplegia type 39 (SPG39; MIM #612020) (OMIM, 2025). These disorders share variable combinations of cerebellar ataxia, upper motor neuron involvement, chorioretinal dystrophy, and hypogonadotropic hypogonadism. Specifically: BNS features cerebellar ataxia, chorioretinal dystrophy and hypogonadotropic hypogonadism. GHS presents with cerebellar ataxia, hypogonadotropic hypogonadism, and brisk reflexes OMCS is defined by trichomegaly, chorioretinal dystrophy, short stature, intellectual disability, and hypopituitarism LMS and SPG39 involve upper motor neuron signs, peripheral neuropathy, and occasionally cognitive impairment and/or cerebellar ataxia (Synofzik et al., 2014). No consistent genotype-phenotype correlations have been established for PNPLA6*-*related disorders. To date, the Human Gene Mutation Database (HGMD) documents 109 pathogenic *PNPLA6* variants (https://www.hgmd.cf.ac.uk/ac/gene.php?gene=PNPLA6; accessed June 2025). Elucidating PNPLA6 's role in multisystem disorders may yield novel therapeutic insights.

Here, we present a 13-year follow-up of a Chinese patient with OMCS, who was initially diagnosed at age 5 with clinical features of trichomegaly, scalp hair thinning, thickened eyebrows, chorioretinal dystrophy, retinitis pigmentosa, visual impairment, and growth delay. Clinical diagnosis of OMCS was based on these findings; however, genetic confirmation was not achievable due to technical constraints at that time. During the untreated follow-up period, the proband developed progressive chorioretinal dystrophy, retinitis pigmentosa, multiple pituitary hormone deficiencies, and abnormal secondary sexual characteristics. Subsequent genetic testing identified a novel compound heterozygous *PNPLA6* variant, expanding the known genotype-phenotype spectrum of OMCS. This finding enriches the variant database for OMCS, and provides valuable support for research into its multisystem pathogenesis.

## 2 Methods

### 2.1 Data collection

A family affected by OMCS was recruited through the Ningxia Eye Hospital at the People’s Hospital of Ningxia Hui Autonomous Region. The pedigree spanned two generations and included four family members, including one affected individual. Detailed histories, covering family structure, marital status, reproductive outcomes, and medical background, were obtained from the proband and parents. Based on this information, a pedigree chart was constructed. Both comprehensive physical examinations and specialized ophthalmic assessments were performed on the affected individual.

This study received approval from the Ethics Committee of the People’s Hospital of Ningxia Hui Autonomous Region (Approval No. 2021-KJHM-014) and strictly adhered to the principles of the *Declaration of Helsinki*. Informed consent was secured from all participants, including the proband—a special school student with preserved basic daily living skills and without significant functional impairment.

### 2.2 Clinical examination

**FIGURE 1:**
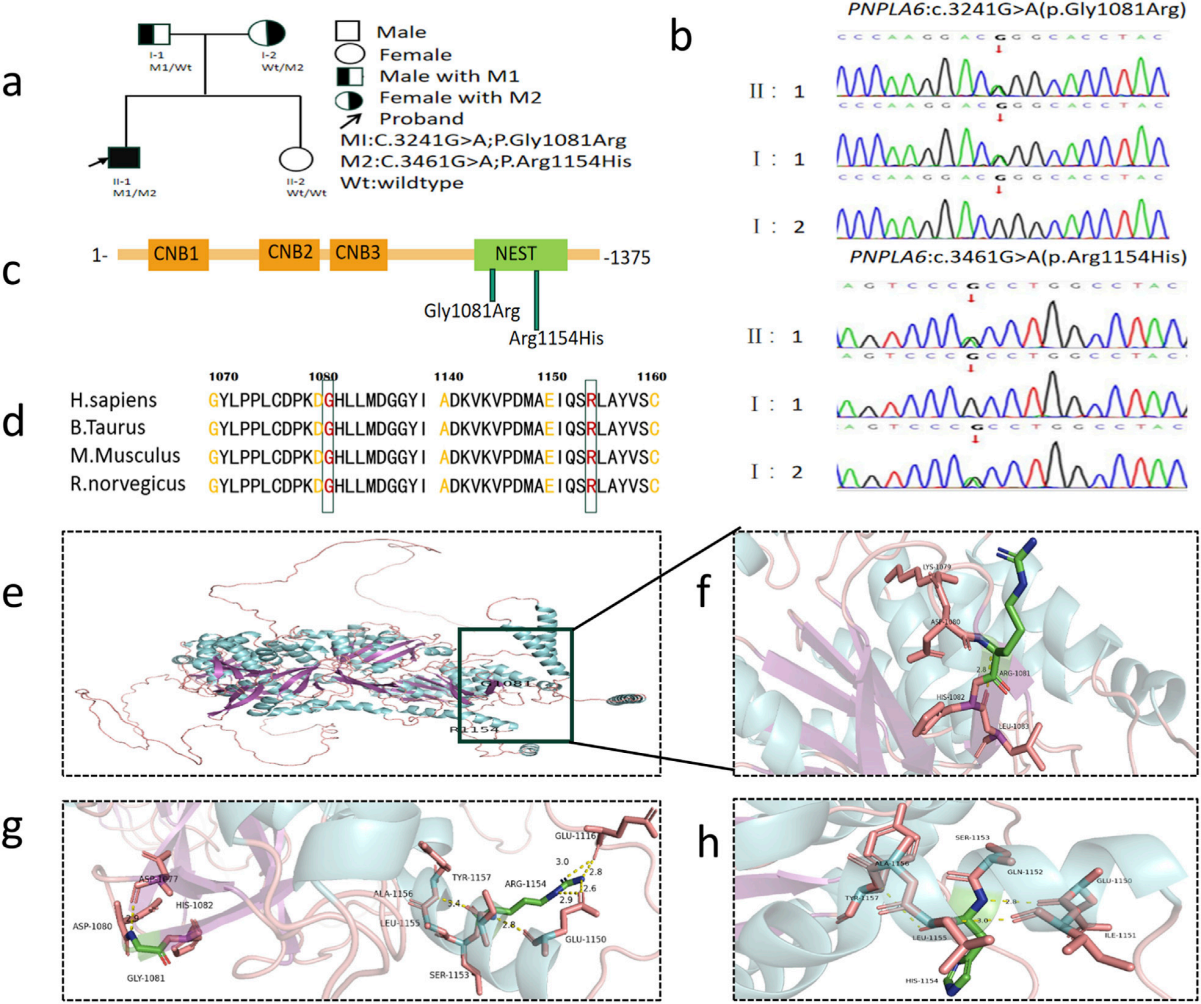
Genetic findings in the family with OMCS. **(a)** Pedigree of the family. The filled black symbols represent the affected member and the arrow denotes the proband. **(b)** By sequencing analysis, compound heterozygous variants of **(c)** 3241G>A and **(c)** 3461G>A were identified in the proband (II:1). **(c)** The PNPLA6 protein structure includes three cyclic nucleotide binding (CNB) domains and a C-terminal patatin-like catalytic domain (NEST). **(d)** The homology of amino acid sequences between human PNPLA6 and other species. The amino acid at position 1,081 (Glycine, Gly1081) and at position 1,154 (Arginine, Arg1154) are highly conserved among species. **(e)** Overall structure diagram of wild-type PNPLA6 protein. **(f)** Local structure diagram of wild-type PNPLA6 protein. **(g)** Gly1081Arg mutation local structure map. **(h)** Arg1154His mutation local structure map.

The proband (Figure 1a, II:1), a 18-year-old male born to non-consanguineous healthy parents (I:1, I:2) with a healthy older sister (II:2), underwent comprehensive clinical and ophthalmological evaluation. The clinical assessment included cranial magnetic resonance imaging (MRI), hand radiography, Denver Developmental Screening Test, and laboratory analyses (complete blood count, fasting glucose, hepatic/renal function, lipid profile, hepatitis B screening, and comprehensive endocrine panel). Ophthalmological examinations comprised: best-corrected visual acuity (BCVA) assessment using a Snellen chart and VT-10 autorefractor (Topcon, Japan); intraocular pressure (IOP) measurement; slit-lamp examination; axial length measurement (IOL Master 500, Carl Zeiss Meditec AG); color fundus photography (TRC-NW300; Topcon, Japan); full-field electroretinography (ff-ERG) with corneal jet electrodes (RetiPort ERG; Roland Consult, Germany) following the standards of the International Society of Clinical Electrophysiology of Vision (ISCEV); visual fields (VF) testing (Humphrey Field Analyzer 750i, Carl Zeiss Meditec, United States; 30-2 protocol: 30°field, 76-point grid, 31.5 asb background); and Spectral-domain optical coherence tomography (SD-OCT; Cirrus HD-OCT4000, Carl Zeiss Meditec, United States) with macular cube (512 × 128) and HD 5-line raster scans through the foveal center. Bilateral mydriasis was induced using 0.5% tropicamide ophthalmic solution prior to posterior segment imaging.

### 2.3 Genetic testing and analysis

WES was performed on peripheral blood samples from the proband and family members. Genomic DNA was extracted, and libraries prepared. Target regions, including exons, adjacent splice sites (±20 bp), and the mitochondrial genome, were captured and enriched via probe hybridization. Following quality control, high-throughput sequencing was conducted. Quality-filtered reads were aligned to the hg38 human reference genome using Bio-BWA software. Single nucleotide variants (SNVs) and insertions/deletions (InDels) were called using GATK haplotypeCaller, then annotated and filtered using clinical databases and bioinformatics tools. Variant interpretation adhered to the guidelines of the American College of Medical Genetics and Genomics (ACMG) and the recommendations of the ClinGen Sequence Variant Interpretation (SVI) working group (Richards et al., 2015). Co-segregation analysis was performed via Sanger sequencing in family members.

### 2.4 In silico analysis

Identified variants were screened against the HGMD to check for previously reported pathogenicity. Pathogenicity assessment followed ACMG guidelines, classifying variants into five categories: pathogenic, likely pathogenic, uncertain significance, likely benign and benign. Evolutionary conservation was analyzed using UniProt. Computational pathogenicity predictions were generated using PolyPhen-2, MutationTaster, and FATHMM-MKL. The wild-type PNPLA6 protein structure was modeled using SWISS-MODEL, with mutant conformations visualized in PyMol.

## 3 Results

### 3.1 History and clinical manifestations of the proband

**FIGURE 2:**
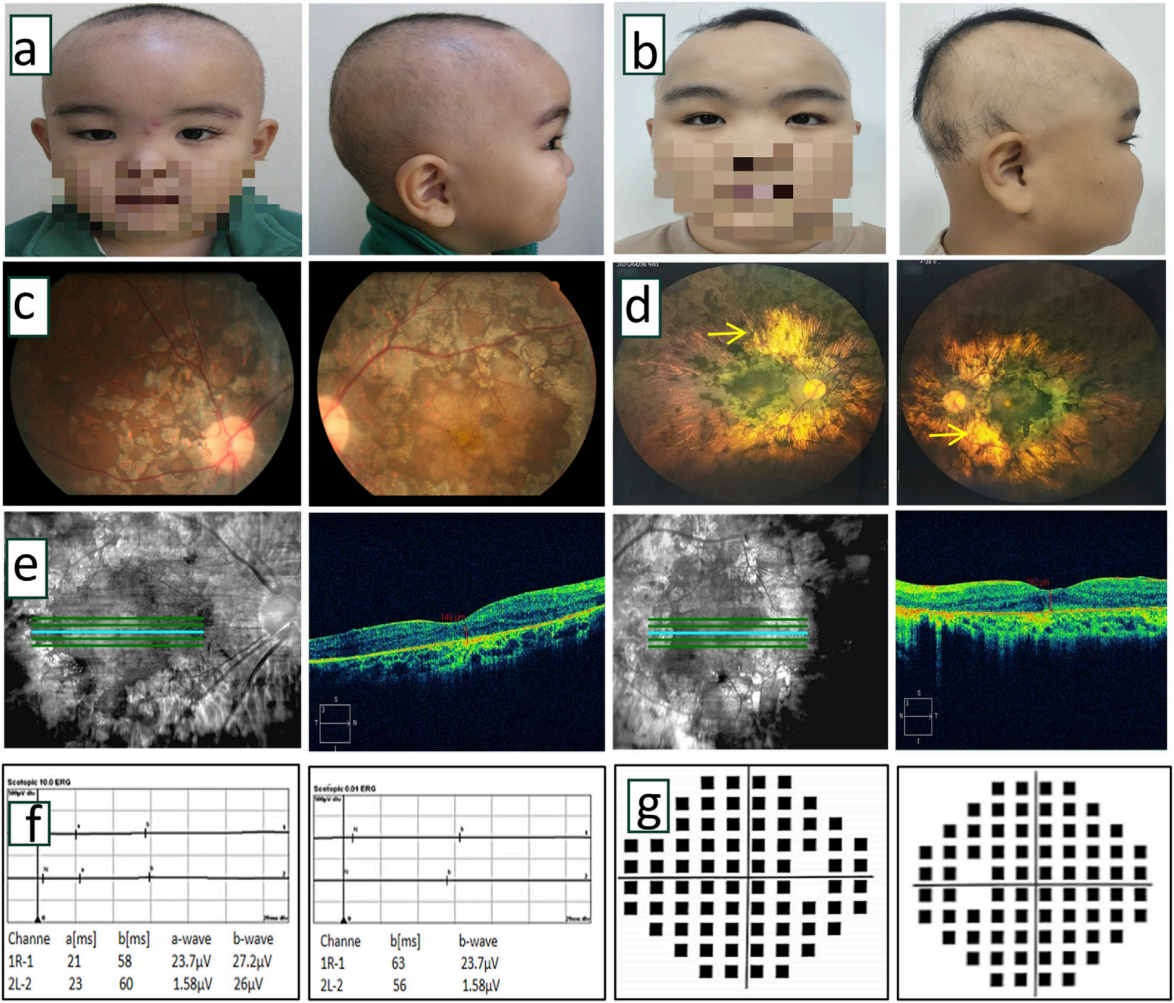
Clinical examination of proband. **(a,b)** Front and side photos of the proband at 5 years old and 18 years old. The proband exhibited sparse hair, thick eyebrows, and long, upward-curled eyelashes (trichomegaly). **(c)** Color fundus photographs of the proband at the age of 5 years indicated obvious chorioretinal dystrophy with retinitis pigmentosa in both eyes. **(d)** The laser scanning fundus images of the proband after 13 years of follow-up showed a clear boundary of the optic disc in both eyes, light red color, no obvious optic cup, extensive uneven chorioretinal dystrophy with retinitis pigmentosa, and partial porcelain white sclera visible in the posterior pole (→ indicated). **(e)** OCT scanning images of the proband at 13 years follow-up indicated thinning of retinal tissue structure, absence of ellipsoid band, uneven reflection signal of retinal pigment epithelium, and uneven degeneration of choroid membrane. **(f)** After 13 years of follow-up, ERG showed that a wave and b wave disappeared in the 10.0 response of ocular dark adaptation, showing an extinction type. Binocular dark adaptation 0.01 showed the disappearance of b wave, which showed the extinction type. **(g)** After 13 years of follow-up, visual field testing showed severe visual field defects in both eyes.

The proband, born to healthy non-consanguineous parents without a family history of stillbirth, intellectual disability, or congenital anomalies, presented to Ningxia Eye Hospital at 5 years of age with night blindness. Neonatal parameters (weight, length, head circumference) were within normal ranges (Li et al., 2009). At the initial assessment, the proband measured 102 cm in height (<10th percentile) and weighed 18.5 kg (between the 25th and 50th percentile). Hand X-ray bone age assessment revealed delayed skeletal maturation. With the proband presenting sparse scalp hair, thick eyebrows, and trichomegaly (Figure 2a) (Sheng et al., 2015), no remarkable abnormalities were observed in the other family members. Cranial MRI showed unremarkable neuroanatomy. Neurological evaluation demonstrated intact motor function, coordination, tendon reflexes, and muscle tone. The Denver Developmental Screening Test indicated minor deficits in gross motor and language domains. Funduscopic examination revealed bilateral retinochoroidopathy with patchy atrophy and retinal pigmentary degeneration (Figure 2c) (Sheng et al., 2015).

**FIGURE 3:**
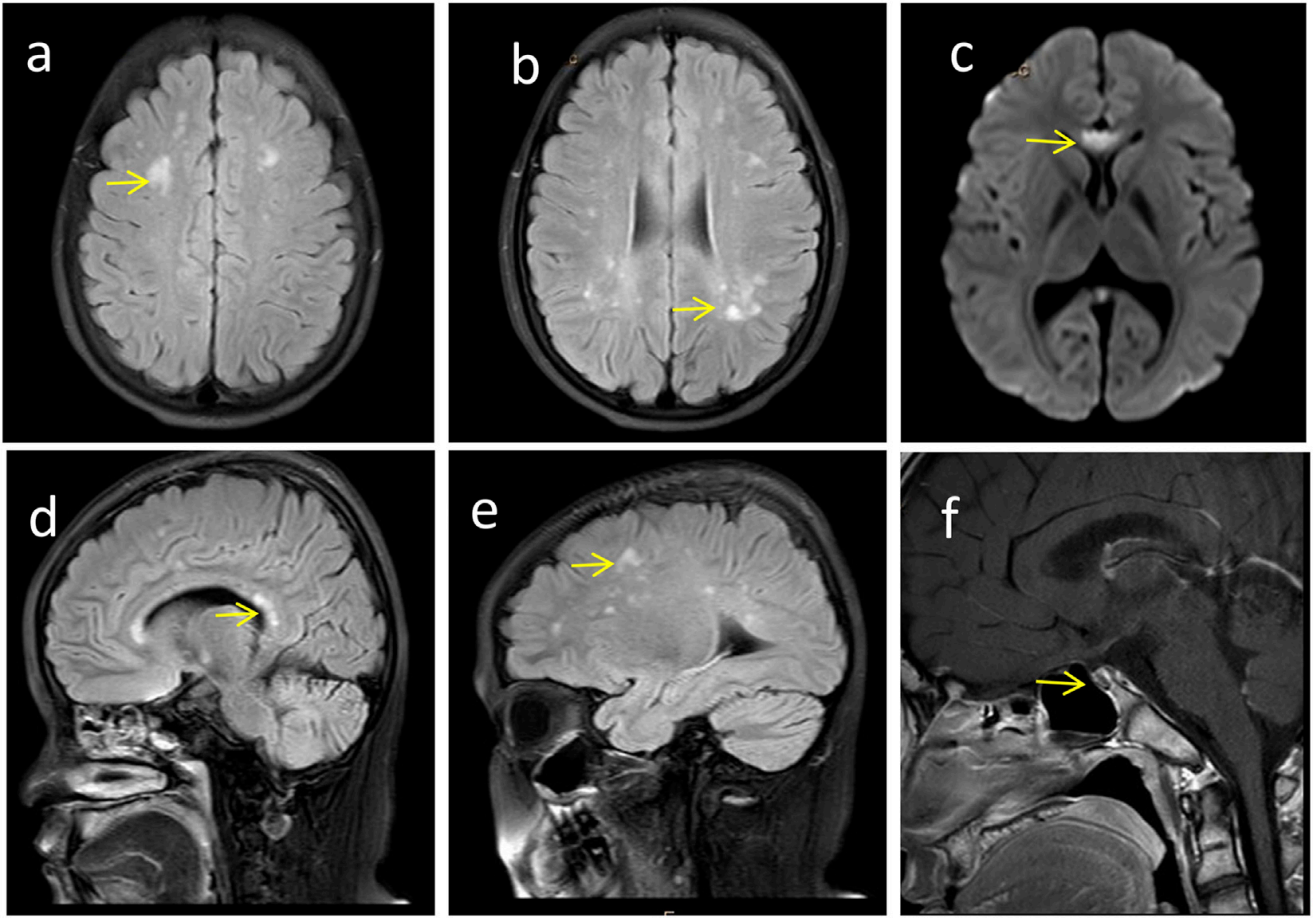
Craniocerebral imaging findings of the proband. **(a-e)** Brain MRI reveals high-signal white matter lesions (→ indicated), which involve the bilateral centrum semiovale, periventricular regions, as well as the genu and splenium of the corpus callosum. **(f)** Enhanced MRI of the pituitary gland demonstrates pituitary atrophy (→ indicated).

Follow-up at 18 years of age, the proband’s height was 158 cm (<10th percentile) and weight was 60 kg (between the 50th and 75th percentile). Physical characteristics included thick eyebrows, sparse scalp hair, trichomegaly, abnormal secondary sexual characteristics (micropenis, absent Adam’s apple, and absence of pubic and axillary hair), gait instability with frequent falls, dyspraxia (inability to carry basins without spilling), and impaired coordination (inability to maintain a single-leg stance) (Figure 2b). The neurological physical examination revealed no obvious abnormalities. However, a comprehensive neurophysiological assessment (including electroencephalogram, and visual evoked potentials) could not be completed as the patient and the family declined these tests. Cranial MRI (Figures 3a–e) showed T2-WI/T2-FLAIR hyperintense white matter lesions (Fazekas Ⅱ) in the bilateral centrum semiovale, periventricular regions, and genu/splenium of the corpus callosum. Callosal lesions appeared slightly hypointense on T1WI and hyperintense on DWI. Pituitary MRI with contrast (Figure 3f) revealed a sagittal height of 2.5 mm, consistent with pituitary atrophy.

Laboratory investigations revealed significant abnormalities in endocrine parameters. Hormonal assays showed decreased levels of LH, GH, prolactin (PRL), progesterone (P), testosterone (T), and free thyroxine (FT4), along with elevated insulin levels. Additionally, hematological and metabolic abnormalities were observed, including an abnormal red cell distribution width (RDW), derangements in the complete blood count (white blood cell count [WBC], hematocrit), hyperuricemia, abnormal liver function (aspartate aminotransferase [AST], AST/ALT ratio) and lipid profile abnormalities (elevated triglycerides, low high-density lipoprotein cholesterol [HDL-C].

Ophthalmic evaluation revealed bilateral high myopia with BCVA of 0.01 (with a −6.00 spherical equivalent refractive correction) in oculus dexter (OD) and 0.05 (with a −6.25 spherical equivalent refractive correction) in oculus sinister (OS). Axial length measurements were 23.10 mm (OD) and 23.11 mm (OS). He showed bilateral horizontal nystagmus without obvious squint. Slit-lamp biomicroscopy showed normal anterior segment. Fundoscopy revealed well-defined, light red optic discs with absent cupping, extensive patchy chorioretinal dystrophy with retinitis pigmentosa, and posterior pole exposure of porcelain-white sclera (Figure 2d). OCT demonstrated retinal thinning, loss of the ellipsoid zone, and irregular chorioretinal atrophy (Figure 2e). VF testing showed severe bilateral constriction (Figure 2g). ff-ERG showed extinguished waveforms (absent a- and b-waves), confirming severe chorioretinal dystrophy (Figure 2f).

### 3.2 Genetic test results

WES identified compound heterozygous variants in the *PNPLA6* gene of the proband (Figure 1a, II:1): a known missense variant (M1) and a novel missense variant (M2) (Figure 1a). The PNPLA6 protein structure contains three cyclic nucleotide binding (CNB) domains and a C-terminal patatin-like catalytic domain (NEST). Both M1 and M2 mutations are located within the NEST (Figure 1c). Sanger sequencing confirmed the segregation pattern: the proband and father (I:1) carried the M1 variant, while the proband and mother (I:2) harbored the M2 variant (Figure 1b). At base pair 3,241 of the *PNPLA6* gene, the M1 variant results in a guanine (G) to adenine (A) substitution, leading to the replacement of glycine (Gly) with arginine (Arg) at position 1,081 in the protein-coding sequence. This disrupts the original hydrogen bond between Gly1081 and Asp1080, increasing positive charge and reducing hydrophobicity at residue 1,081. Similarly, at base pair 3,461, the M2 variant involves a guanine (G) to adenine (A) substitution, replacing arginine (Arg) with histidine (His) at position 1,154 in the protein-coding sequence. This abolishes the hydrogen bond between Arg1154 and Glu1116, decreasing positive charge and enhancing hydrophobicity at residue 1,154 (Figures 1e–h). The M1 variant has a population frequency of 0 in the gnomAD_EAS database, while M2 is absent from gnomAD and unreported in the literature or genomic databases, supporting its novelty. Phylogenetic conservation analysis revealed high evolutionary conservation at residues 1,081 and 1,154 across species (Figure 1d), indicating critical roles in PNPLA6 function. Both variants were bioinformatically predicted to be deleterious (Table 1). According to ACMG guidelines, M1 was categorized as a likely pathogenic (LP = PS3+PM3+PM2+PP3+PP4), and M2 as likely pathogenic (LP = PM2+PM3+PP3+PP4) (Table 2). Collectively, the compound heterozygous variants in the *PNPLA6* gene likely disrupt protein structure and function, contributing to the pathogenesis of OMCS.

**TABLE 1:** The effects of *PNPLA6* variations on their protein function by *in silico* analysis.

Variants	Software	Score	Predicted signal
c.3241G>A (p.Gly1081Arg)	Mutation taster	1.0	Disease causing
c.3461G>A (p.Arg1154His)	Mutation taster	1.0	Disease causing
c.3241G>A (p. Gly1081Arg)	PolyPhen-2	1.0	Damaging
c.3461G>A (p. Arg1154His)	PolyPhen-2	1.0	Damaging
c.3241G>A (p. Gly1081Arg)	fathmm-MKL	0.99199	Damaging
c.3461G>A (p. Arg1154His)	fathmm-MKL	0.99553	Damaging

**TABLE 2:** Pathogenicity analysis of variants by ACMG.

Variants of *PNPLA6*	Evidence of pathogenicity
NM_006702,5: exon30: c.3241G>A	a. Functional studies have been supportive of a damaging effect on the gene or gene product for this variant. (PS3_Moderate). b. It has been reported that pathogenic variant p.R1031fs+38 has been detected in the trans of this variant (PM3). c. This variant is a rare variant with a frequency of 0 in the general East Asian population in gnomAD database (PM2_Supporting). d. Multiple bioinformatics software predicted deleterious effects of the mutation on genes or gene products (PP3) e. Proband’s proband’s phenotype is highly specific for Oliver-McFarlane syndrome with a single genetic etiology (PP4)
NM_006702.5: exon31: c.3461G>A	a. This mutation is a rare variation and was not included in the general East Asian population in gnomAD database. (PM2_Supporting) b. This variant was located in the trans of the likely pathogenic variant M2 (c.3461G>A: p. Arg1154His) (PM3). c. Multiple bioinformatics software predicts harmful effects of the variant on genes or gene products (PP3) d. proband’s phenotype is highly specific for Oliver-McFarlane syndrome with a single genetic etiology (PP4)

ACMG, Standards and Guidelines for Interpretation of Sequence Variants issued by American College of Medical Genetics and Genomics (ACMG) in 2015.

## 4 Discussion

### 4.1 The evolution of clinical manifestations in this case of OMCS

We present a 13-year longitudinal follow-up of an OMCS case with novel genetic associations. Clinical diagnosis was established at age five based on characteristic features: chorioretinal dystrophy, retinitis pigmentosa, growth delay, trichomegaly (presenting as elongated curly eyelashes and thick eyebrows), and sparse scalp hair. Subsequent monitoring revealed persistent developmental delays, short stature, and gross motor dysfunction. Without therapeutic intervention, retinochoroidopathy progressively worsened, leading to a decreased BCVA. Concurrently, the patient developed endocrine deficiencies during adolescence, including absence of secondary sexual characteristics. Laboratory analysis confirmed deficiencies in LH and GH, while FSH and TSH levels remained within normal ranges. Neuroimaging confirmed pituitary atrophy.

### 4.2 Clinical phenotypic features of OMCS

First described in 1965, OMCS is a rare autosomal recessive disorder caused by pathogenic variants in the *PNPLA6* gene. Its core clinical manifestations include trichomegaly, severe chorioretinal dystrophy, and combined pituitary hormone deficiencies (GH, TSH, LH/FSH) (Patsi et al., 2018; Liu et al., 2020). Our systematic review of 36 published cases (Table 3) showed that trichomegaly (35/36,97%) and chorioretinal dystrophy (36/36,100%) are universal features, while GH deficiency (25/36, 69%), gonadotropin deficiency (24/36,67%), delayed bone age (27/36, 75%), and hair abnormalities (26/36, 72%) are highly prevalent. Approximately half of the patients exhibit TSH deficiency (16/36,44%), and neurological symptoms (21/36,58%). Collectively, these findings establish trichomegaly, chorioretinal dystrophy, GH deficiency, and gonadotropin deficiency as cardinal features of OMCS.

**TABLE 3:** Characteristics of Oliver-McFarlane cases previously described in the literature.

Author	Sex	Birth weight(g)	Bone age	Trichomegaly	Retinopathy	Hair	GH deficiency	Hypothyroidism	Hypogonadism	Mental retardation	Neurological involvement	Variation 1	Variation 2
Oliver and McFarlane. (1965)	M	1800	Delayed	+	+	Sparse	—	+	—	+	NA	NA	NA
Cant	F	2500	Delayed	+	+	Alopecia	+	—	+	—	Neuropathy	NA	NA
Sampson et al. (1989)	F	2500	Delayed	+	+	Alopecia	+	—	+	—	Neuropathy	NA	NA
Corby et al. (1971)	M	2000	Delayed	+	+	Sparse	—	—	—	+	NA	NA	NA
Zaun et al. (1984)	F	1700	Delayed	+	+	Alopecia	—	—	—	—	—	NA	NA
Zaun et al. (1984)	M	2600	Delayed	+	+	Sparse	—	+	+	+	Ataxia	NA	NA
Patton et al. (1986)	M	NA	Delayed	+	+	Alopecia	+	+	+	Mild	Ataxia,spastic,paraplegia, Neuropathy	p.(Arg983Glnfs*38) c.2944_2947dupAGCC	p.(Gly1081Arg) c.3241G>A
Hufnagel et al. (2015)	M	NA	Delayed	+	+	Alopecia	+	+	+	Mild	Ataxia,spastic,paraplegia, Neuropathy	p.(Arg983Glnfs*38) c.2944_2947dupAGCC	p.(Gly1081Arg) c.3241G>A
Matheieu et al. (1991)	M	3110	Delayed	+	+	Alopecia	—	—	+	Mild	Peripheral	NA	NA
Matheieu et al. (1991)	M	2400	Delayed	+	+	—	+	—	+	—	neuropathy	NA	NA
Haritoglou et al. (2003)	M	2220	Delayed	+	+	Sparse	NA	—	+	+	—	NA	NA
Haimi and Gershoni—Baruch. (2005)	M	1560	Delayed	+	+	Sparse	+	—	+	Mild	Ataxia, neuropathy	NA	NA
Haimi and Gershoni—Baruch. (2005)	F	1951	Delayed	+	+	Sparse	+	—	—	Mild	—	NA	NA
Somnez et al. (2008)	M	2360	NA	+	+	—	+	+	+	+	Neuropathy	NA	NA
Luiz Pedroso et al. (2014)	F	NA	NA	+	+	Alopecia	NA	NA	+	NA	Ataxia	NA	NA
Kmoch et al. (2015)	F	2000	Delayed	+	+	—	+	+	—	—	—	*p.(Leu476Pro) c.1427T>C*	*p.(Asp1077Asn) c.3229G>A*
Kmoch et al. (2015)	M	NA	NA	+	+	Sparse	+	+	—	—	Neuropathy and/or ataxia	p.(Gln658*) c.1972C>T	*p.(Gly1081Arg) c.3241G>C*
Kmoch et al. (2015)	F	3000	NA	+	+	Alopecia	+	—	+	—	Neuropathy and/or ataxia	*c.199–2A>T*	*p.(Asp1077Asn) c.3229G>A*
Kmoch et al. (2015)	F	3300	NA	+	+	—	+	—	+	—	Neuropathy and/or ataxia	*c.199–2A>T*	*p.(Asp1077Asn) c.3229G>A*
Kmoch et al. (2015)	M	1900	NA	+	+	Alopecia	+	—	+	—	Neuropathy and/or ataxia	*c.199–2A>T*	*p.(Asp1077Asn) c.3229G>A*
Kmoch et al. (2015)	F	2500	Delayed	+	+	Alopecia	+	+	+	Mild	Neuropathy and/or ataxia	p.(Arg1060Trp) c.3178C>T	p.(Gly1081Arg) c.3241G>C
Kmoch et al. (2015)	F	2500	Delayed	—	+	Alopecia	+	+	+	—	Neuropathy	p.(Leu366Serfs*28) c.1094dup	p.(Gly1081Arg)c.3241G>C
Kmoch et al. (2015)	M	2400	Delayed	+	+	NA	+	+	+	+	Neuropathy and/or ataxia	p.(Trp873*) c.2619G>A	NA,only one variant
Hufnagel et al. (2015)	F	2555	Delayed	+	+	Sparse	+	+	NA	—	—	p.(Arg1051Gln) c.3152G>A	p.(Gly1128Ser) c.3382G>A
Hufnagel et al. (2015)	M	2612	Delayed	—	+	Sparse	+	+	NA	—	—	p.(Arg1051Gln) c.3152G>A	p.(Gly1128Ser) c.3382G>A
Hufnagel et al. (2015)	M	NA	Delayed	+	+	—	+	+	NA	—	Neuropathy	p.(Arg983Glnfs*38) c.2944_2947dupAGCC	p.(Gly1081Arg) c.3241G>A
Hufnagel et al. (2015)	M	NA	NA	+	+	Alopecia	+	—	+	—	—	c.1829+2T>G	p.(Val1167Ala) c.3500T>C
Hufnagel et al. (2015)	M	NA	Delayed	+	+	—	+	+	NA	—	—	dup(ex14_20)	p.(Val1167Ala) c.3500T>C
Patsi et al. (2018)	F	NA	NA	+	+	Sparse	+	+	+	NA	Ataxia, neuropathy	p.(Ser1159Tyr) c.3476C>A	p.(Ser1159Tyr) c.3476C>A
Makuloluwa et al. (2020)	F	2980	Delayed	+	+	—	—	—	—	—	—	*p.(Ala1064Thr) c.3190G>A*	*p.(Arg1135Trp) c.3403C>T*
Liu et al. (2020)	M	1900	NA	+	+	NA	NA	+	+	+	Seizures	*p.(Gln497His) c.1491G>T*	*p.(Gly1123Arg) c.3367G>A*
Wu et al. (2021)	M	NA	Delayed	+	+	Sparse	+	NA	+	—	—	*p.(Ser997Leu) c.2990C>T*	*c.3702 + 1G>A*
Kristian et al	F	1650	Delayed	+	+	Sparse	+	—	NA	—	—	*p.(Arg277Pro) c.830G>C*	*p.(Gly1081Arg) c.3241G>A*
Kristian et al	M	1430	Delayed	+	+	Sparse	+	—	+	—	—	p.(Leu366Serfs*28) c.1094dup	*p.(Asp1147Glu) c.3441C>G*
Shi et al. (2023)	F	NA	Delayed	+	+	—	NA	—	+	NA	Neuropathy and/or ataxia	*p.(Asp1141Gly) c.3342A>G*	p.(Phe338Leufs*80) c.1094dup
Our case	M	2950	Delayed	+	+	Alopecia	—	—	+	Mild	—	*p.(Gly1081Arg) c.3241G>A*	*p.(Arg1154His) c.3461G>A*

M, malen; F, female, +: Present, –: not presen; NA, informaton not available; GH, growth hormone.

### 4.3 Trichomegaly in OMCS

Trichomegaly-characterized by abnormal lengthening, thickening, curling, or pigmentation of eyelashes-is a hallmark of OMCS. Although eyelash trichomegaly may be drug-induced, surgery-related, or associated with other conditions, its pathogenesis in OMCS is thought to involve dysregulated hair follicle biology (Hutchison et al., 2022). Epidermal growth factor receptors (EGFR), highly expressed in the proliferative, undifferentiated outer root sheath of hair follicles, play a crucial role in hair growth, as evidenced by the hair growth inhibition observed with EGFR antagonists. Additionally, prostaglandins can transition hair follicles from telogen (resting) to anagen (growth) phase (Lacouture et al., 2006; Dueland et al., 2003). Studies suggest that trichomegaly is associated with elevated levels of phosphatidylethanolamine in follicular cells, which promotesincreased prostaglandin production and abnormal eyelash growth (Yamamoto et al., 2011). However, the exact mechanisms in OMCS remain incompletely understood.

### 4.4 Chorioretinal dystrophy

Chorioretinal dystrophy is a core feature of *PNPLA6-*related OMCS, presenting as progressive vision loss, visual field defects, and characteristic fundus changes, typically with onset before 5 years of age. Funduscopy reveals diffuse chorioretinal atrophy and pigment clumping, OCT demonstrates retinal thinning, loss of the layered architecture, RPE depletion, and choriocapillaris loss (Doğan et al., 2021). In our proband, persistent choroidal and retinal atrophy was observed. The pathophysiology may involve the loss of phospholipase B (PLB) activity caused by *PNPLA6* variants. PNPLA6 facilitates choline transfer from RPE cells to photoreceptors, thereby supporting their survival. An impaired choline supply leads to abnormal morphology, proliferation, metabolism, and function of RPE and photoreceptor cells, contributing to visual deterioration and retinal degeneration (Ono et al., 2025). Although no validated treatment has been reported to reverse retinal damage, early intervention aimed at preserving RPE and photoreceptor function may be beneficial, given the early onset and progressive nature of retinal involvement.

### 4.5 Pituitary hormone deficiencies and neurological manifestations

Combined pituitary hormone deficiencies contribute to developmental delays and abnormal secondary sexual characteristics in OMCS. GH/LH deficiencies, as seen in our proband, can lead to impaired pubertal development, including micropenis, small testes, sparse facial hair, high-pitched voice, and lack of voice breaking (Nedresky and Singh, 2022). In this study, low GH and LH levels, compounded by the patient’s refusal of hormone replacement therapy, may have exacerbated growth retardation and abnormal sexual development.

Neurological and imaging abnormalities are also reported in *PNPLA6*-related disorders, including white matter signal changes, pituitary and cerebellar atrophy, and empty sella turcica. These may arise from loss of NTE function, leading to endoplasmic reticulum damage, vacuolation of nerve cell bodies, and abnormal reticular aggregates in the nervous system. NTE plays a crucial role in maintaining intracellular phospholipid homeostasis. Therefore, *PNPLA6* variants may cause neurodegeneration via NTE dysfunction (Topalogl et al., 2014; Akassoglou et al., 2004). In our proband, cranial MRI showed hyperintense white matter signals involving the bilateral centrum semiovale, periventricular areas, and corpus callosum (genu and splenium), along with pituitary atrophy. White matter changes may underlie gait and balance impairments, while pituitary atrophy likely contributes to hormone deficits.

Multidisciplinary evaluation (ophthalmology, neurology, endocrinology) and genetic testing improve diagnostic accuracy for OMCS. Although no consensus on a standard treatment approach has been established, early hormone replacement therapy may mitigate developmental and intellectual impairments. Emerging strategies, including gene therapy, local choline application, and NTE activity modulation, show promise but require further validation (Shi et al., 2023; Liu et al., 2024).

### 4.6 Gene structure and function

PNPLA6 (Patatin-like phospholipase domain containing 6), originally termed NTE, is a member of the nine-protein patatin-like phospholipase family. Located on chromosome 19p13.2, the *PNPLA6* gene comprises 37 exons and produces five transcript variants; the longest (NM_001166111) encodes a 1,375-amino-acid protein (151 kDa) with three domains: an N-terminal transmembrane domain, three cyclic nucleotide binding (CNB) domains, and a C-terminal patatin-like catalytic domain (NEST) (Liu and Hufnagel, 2023). Among 109 reported *PNPLA6* variants (HGMD), missense/nonsense variants are most common (84/109, 77%), followed by splicing (10/109, 9.2%), small insertions/deletions/indels (12/109, 11%), and gross insertions/deletions (3/109, 2.8%). Over 50% localize to the patatin domain; missense variants within the NEST domain correlate with severe retinopathy and endocrinopathy (Liu et al., 2024).

*PNPLA6* variants are linked to five clinically heterogeneous disorders: BNS, OMCS, GHS, LMS and SPG39. Although comorbid spastic paraplegia and parkinsonism have been reported in *PNPLA6*-related disorders, the proband in this study has not yet developed these signs, exhibiting only mild gait instability, ataxia, and coordination disorders (Kazanci et al., 2022). Given the proband’s current young age, this absence could b age-dependent, which warrants long-term follow-up to monitor disease progression. Overlapping features include chorioretinopathy (in BNS, OMCS, and LMS) and cerebellar ataxia with hypogonadotropism (in GHS and SPG39) (Table 4). No correlation exists, but disease severity inversely correlates with residual NTE activity (Liu et al., 2024).

**TABLE 4:** *PNPLA6* Disorders: Comparison of Phenotypic Clusters by Select Features. OMCS = Oliver-McFarlane syndrome; BNS = Boucher-Neuhäuser syndrome; GHS = Gordon Holmes syndrome; LMS = Laurence-Moon syndrome; SPG39 = spastic paraplegia type 39. Note: The clusters in this table do not constitute distinct phenotypes, as they may overlap in many affected individuals.

Phenotypic feature	PNPLA6 disorders					
	OMCS	BNS	GHS	LMS	SPG39	Parkinsonian
Cerebellar ataxia		+	+	+	+	
Peripheral neuropathy				+	+	
Cognitive dysfunction					+	
Chorioretinal dystrophy	+	+		+		
Trichomegaly	+					
Hypothyroidism	+	+	+			
Hypogonadism	+	+	+			
GH deficiency	+				+	
Motor dysfunction						+

PNPLA6 is ubiquitously expressed, with high levels in the central nervous system, lymphoid tissue, kidney, lung, and testis. In the eye, it is abundant in RPE cells but minimally expressed in photoreceptors. Functionally, PNPLA6 exhibits lysophospholipase/PLB activity, hydrolyzing lysophosphatidylcholine (LPC) and phosphatidylcholine (PC) to release glycerophosphocholine (GPC), which is further metabolized into choline-critical for cellular homeostasis. In the retina, PNPLA6*-*mediated PC catabolism mobilizes endogenous choline, whose recycling into PC is essential for RPE and photoreceptor integrity (Richardson et al., 2020; Ono et al., 2025). Loss of PNPLA6 activity, due to pathogenic variants, disrupts choline metabolism and triggers retinal degeneration via two key pathways: ① Accumulation of PC and LPC in RPE cells, coupled with reduced choline, impairs proliferation, adhesion, phagocytosis, and mitochondrial dysfunction; ② Disrupted choline secretion from RPE to photoreceptors leads to structural and functional impairment of photoreceptors due to choline deficiency. Notably, choline supplementation reverses retinal pathology in *PNPLA6-*deficient models: local administration of 2% choline restored retinal thickness, photoreceptor outer segment structure, and enhanced visual function in *PNPLA6-*deficient mice (Ono et al., 2025). This suggests choline modulation as a promising therapeutic strategy for *PNPLA*6-associated retinopathy, though further research is needed.

While animal studies have established that *PNPLA6* variants impair NTE activity and disruptcholine metabolism to cause disease, the complete pathogenic cascade remains incompletely elucidated. Key unanswered questions include how these variants progressively compromise protein function and disrupt core cellular processes-such as signal transduction, cell cycle, cell death, and cell differentiation and how these disruptions ultimately manifest as multisystem abnormalities including chorioretinal dystrophy, neurological disorders, and endocrine dysfunction (Liu et al., 2024; Richardson et al., 2020). Future research should integrate systematic clinical follow-up with in-depth basic science to delineate the specific molecular steps and regulatory networks through which *PNPLA6* gene variants impair NTE activity and choline metabolism. Such efforts are crucial for deciphering diseases pathogenesis and will lay the necessary theoretical foundation for the developing targeted therapies.

## 5 Conclusion

This study identifies a novel compound heterozygous *PNPLA6* variant (c.3241G>A/c.3461G>A) in an OMCS patient, expanding the spectrum of pathogenic variants and clinical phenotypes. Long-term follow-up confirms the progressive nature of OMCS, particularly affecting RPE and photoreceptor cells and leading to chorioretinal atrophy. Early intervention aimed at preserving RPE and photoreceptor function may be beneficial, given the early onset and progressive nature of retinal involvement.

## Data Availability

The datasets for this article are not publicly available due to concerns regarding participant/patient anonymity. Requests to access the datasets should be directed to the corresponding authors.

## Glossary

OMCS: Oliver-McFarlane syndrome
WES: whole-exome sequencing
RPE: retinal pigment epithelium
GH: growth hormone
LH: luteinising hormone
FSH: follicle-stimulating hormone
TSH: thyroid-stimulating hormone
NTE: neuropathy target esterase
BNS: Boucher-Neuhauser syndrome
GHS: Gordon-Holmes syndrome
OMCS: Oliver-McFarlane syndrome
LMS: Lawrence-moon syndrome
SPG39: spastic paraplegia type 39
HGMD: Human Gene Mutation Database
MRI: magnetic resonance imaging
BCVA: best-corrected visual acuity
ff-ERG: full-field electroretinography
M1: (c.3241G>A, p. Gly1081Arg)
M2: (c.3461G>A, p. Arg1154His)
VF: visual fields
OCT: optical coherence tomography
ACMG: College of Medical Genetics and Genomics
OD: oculus dexter; OS, oculus sinister
A: adenine
Gly: glycine
Arg: arginine
His: histidine
EGFR: Epidermal growth factor receptors
LPC: lysophosphatidylcholine
PC: phosphatidylcholine
GPC: glycerophosphocholine
